# miR-2861 acts as a tumor suppressor via targeting EGFR/AKT2/CCND1 pathway in cervical cancer induced by human papillomavirus virus 16 E6

**DOI:** 10.1038/srep28968

**Published:** 2016-07-01

**Authors:** Junfen Xu, Xiaoyun Wan, Xiaojing Chen, Yifeng Fang, Xiaodong Cheng, Xing Xie, Weiguo Lu

**Affiliations:** 1 Department of Gynecologic Oncology, Women’s Hospital, School of Medicine, Zhejiang University, Hangzhou, Zhejiang 310006, China; 2 Women’s Reproductive Health Laboratory of Zhejiang Province, Women’s Hospital, School of Medicine, Zhejiang University, Hangzhou, Zhejiang 310006, China; 3 Department of General Surgery, Sir Run Run Shaw Hospital, School of Medicine, Zhejiang University, Hangzhou 310016, China

## Abstract

Persistent infection with oncogenic human papillomavirus viruses (HPVs) is a casual factor for cervical cancer and its precursors, and the abnormal constitutive expression of viral oncoprotein E6 is a key event during the malignant transformation. Here, we performed miRNA microarray to identify changes of miRNAs following ectopic HPV16 E6 overexpression in HEK293T cells and found miR-2861 was greatly decreased in both HEK293T and HaCaT cells expressing HPV16 E6 compared to vector control. Further, we demonstrated a biological link among HPV16 E6, miR-2861, EGFR, AKT2, and CCND1 in cervical cancer cells. We showed that miR-2861 was downregulated in cervical cancer tissues and negatively correlated with advanced tumor stage and lymph node metastasis. Overexpression of miR-2861 suppressed cervical cancer cell proliferation and invasion and enhanced apoptosis. Subsequent investigation revealed that EGFR, AKT2, and CCND1 were all the direct targets of miR-2861. Importantly, silencing EGFR, AKT2, and/or CCND1 recapitulated the cellular effects seen upon miR-2861 overexpression. Restoration of EGFR, AKT2, and/or CCND1 counteracted the effects of miR-2861 expression. Thus, we identified a new pathway employing miR-2861, EGFR, AKT2, and CCND1 that may mediate HPV16 E6 induced initiation and progression of cervical cancer.

Cervical cancer is one of the most common malignancies in women worldwide, with estimated 529,000 new cases and 275,000 deaths each www.who.int/hpvcentre). Persistent infection by oncogenic human papillomaviruses (HPVs) is widely recognized as the leading cause of cervical cancer^1^. Among more than 120 HPV types, only a small subset, termed as high risk (HR-HPV), are cancer-causing; of these, HPV16 and 18 infection are most frequent^2^, and HPV16 has been detected in up to 50% of cervical cancer cases^3^,^4^. It has been known that the persistent over-expression of viral oncoproteins, E6 and E7, directly contribute to cervical carcinogenesis. The p53 and retinoblastoma (RB) proteins are well-characterized host targets of viral E6 and E7. However, recent studies have shown that the alteration of additional targets play equally important roles^5^,^6^. E6 mutants deficient for degradation of p53 are still be able to immortalize cells and required for its full transforming potential^7^,^8^,^9^, suggesting that E6 interactions with other cellular factors are necessary for cancer initiation and development.

MicroRNAs (miRNAs) are small noncoding regulatory RNAs of 18–25 nucleotides that can negatively regulate mRNA stability and/or inhibit mRNA translation^10^,^11^. It is predicted that miRNAs regulate up to 90% of human genes, which suggests that they may exert profound effects on gene expression in almost every biological process^12^,^13^,^14^. miRNA dysregulation is one of the most important factors contributing to cancer development and has been implicated in virus infection^15^,^16^,^17^,^18^. Cervical cancer, like many other tumor types, displays notably increased or decreased expression of a large number of oncogenic or tumor-suppressive miRNAs^19^,^20^,^21^. Though miRNAs encoded by HPV have not been identified, several host miRNAs are indeed involved in HPV induced cervical cancer initiation and progression, such as miR-424^22^, miR-375^23^, miR-34a^24^,^25^, miR-218^26^, miR-23b^27^, and miR-203^28^. Thus, it is conceivable that cellular miRNAs can participate directly in the carcinogenic procedure induced by HPV oncoproteins.

As HPV16 is by far the most common cancer-related HPV type, in the current study, we sought to determine miRNA expression profiles in response to HPV16 oncoprotein E6 overexpression by microarray analysis to identify specific cellular miRNAs that play biological roles in HPV16 E6 induced cancer. Through microarray data analysis, we focused on miR-2861, which expression was regulated by HPV16 E6 in a p53-independent way. Furthermore, we identified a new pathway employing miR-2861, EGFR, AKT2, and CCND1 that mediates HPV16 E6 induced initiation and progression of cervical cancer.

## Results

### HPV16 E6 oncoprotein regulates the expression of a subset of cellular miRNAs

**Figure 1:**
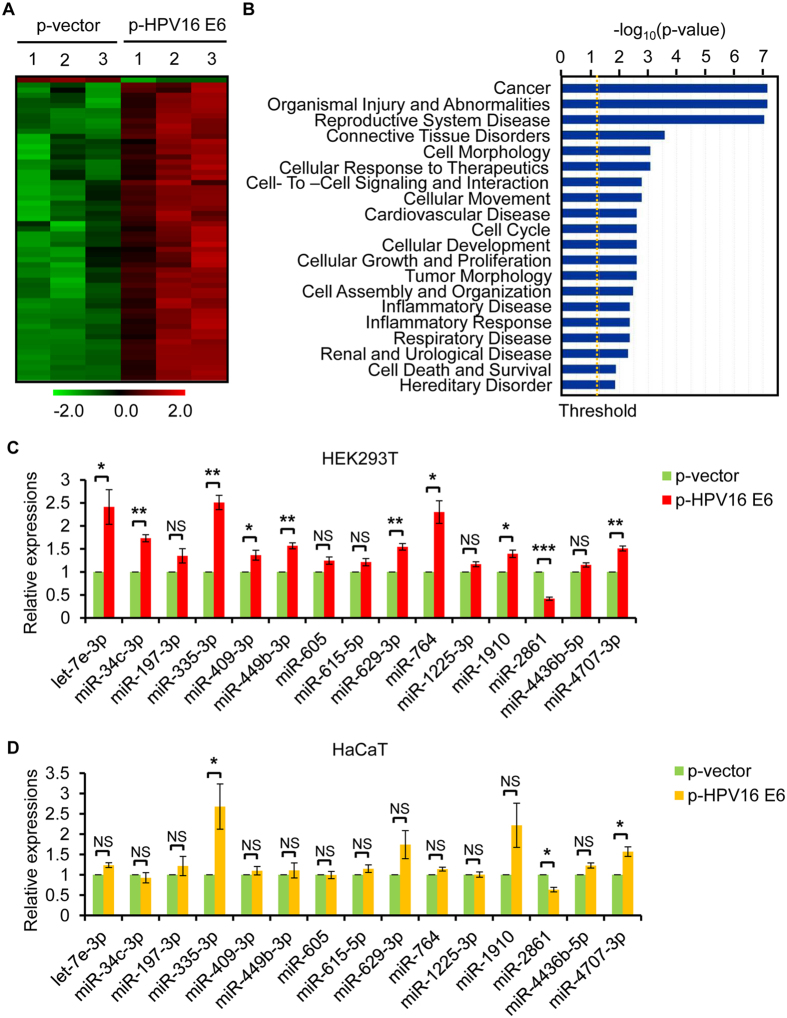
HPV16 E6 regulates the expression of a subset of cellular miRNAs. (**A**) miRNA microarray analysis illustrates expression profiles of miRNAs that are differentially expressed upon HPV16 E6 overexpression in HEK293T cells. Green, decreased expression; Red, increased expression. (**B**) Disease and function analysis (Ingenuity IPA software) of the above 59 differential changes shows the 20 main influenced diseases and functions in HEK293T cells expressing HPV16 E6. (**C**) Verification of selectively 15 altered expression of miRNAs in HEK293T cells following overexpression of HPV16 E6 by qRT-PCR analysis. (**D**) Verification of the altered miRNAs in p53-mutant HaCaT cells expressing HPV16 E6 or empty vector. Error bars represent ± SD of three experiments. *P < 0.05; **P < 0.01; ***P < 0.001; NS, not significant.

To obtain the expression signature of miRNAs in response to HPV16 oncoprotein E6, we performed miRNA microarray analysis using HEK293T cells transfected with HPV16 E6 expression plasmid or empty control plasmid. The transfection level of HPV16 E6 gene was firstly confirmed by qRT-PCR (Supplementary Fig. S1A). As we know that E6 reduces p53 protein level^29^,^30^, we detected p53 protein level to indirectly confirm the expression of HPV16 E6 protein (Supplementary Fig. S1B). Then, we screened miRNA expressions in the two groups, HPV16 E6 expressing and control HEK293T cells, using μParaflo™ microRNA microarray assay (covering Sanger miRBase release 19.0). A total of 59 miRNAs exhibited significantly differential expressions in response to HPV16 E6 overexpression in 293T cells (all *P* value < 0.05 and fold changes ≥ 2) with 58 miRNAs in an up-regulating and 1 in a down-regulating trend (Fig. 1A, Supplementary Table S1).

To determine the biological relevance of the above 59 identified differentially expressed miRNAs, we performed Ingenuity Pathway Analysis (IPA). Disease and function analysis showed that the majority of the miRNAs were associated with cancer (Fig. 1B).

To confirm the microarray findings, 15 miRNAs (let-7e-3p, miR-34c-3p, miR-197-3p, miR-335-3p, miR-409-3p, miR-449-3p, miR-605, miR-615-5p, miR-629-3p, miR-764, miR-1225-3p, miR-1910, miR-2861, miR-4436b-5p, and miR-4707-3p), most of which were not implicated previously in HPV infection and/or HPV-related cancers, were further validated using qRT-PCR (Fig. 1C). The expression trend of these miRNAs is generally consistent with the microarray findings, and 10 of them showed significant difference in HPV16 E6 overexpressed HEK293T cells compared to the control group (*P* value < 0.05). From the viewpoint of novelty, we further validated these 15 miRNAs in p53-mutant HaCaT cells to evaluate the influence of p53 status on HPV16 E6-mediated miRNA dysregulation. The expressions of most of these miRNAs were not changed in HaCaT cells with HPV16 E6 overexpression, consisting with reports that p53 regulates a set of miRNAs^31^,^32^,^33^,^34^. However, there were still 3 miRNAs, miR-335-3p, miR-2861, and miR-4707-3p, showed significant difference (Fig. 1D), indicating HPV16 E6 could regulate these miRNAs independent of p53 status. Taken together, we focused on miR-2861 for further study.

### miR-2861 plays important biological roles in HPV16 positive cervical cancer cells

**Figure 2:**
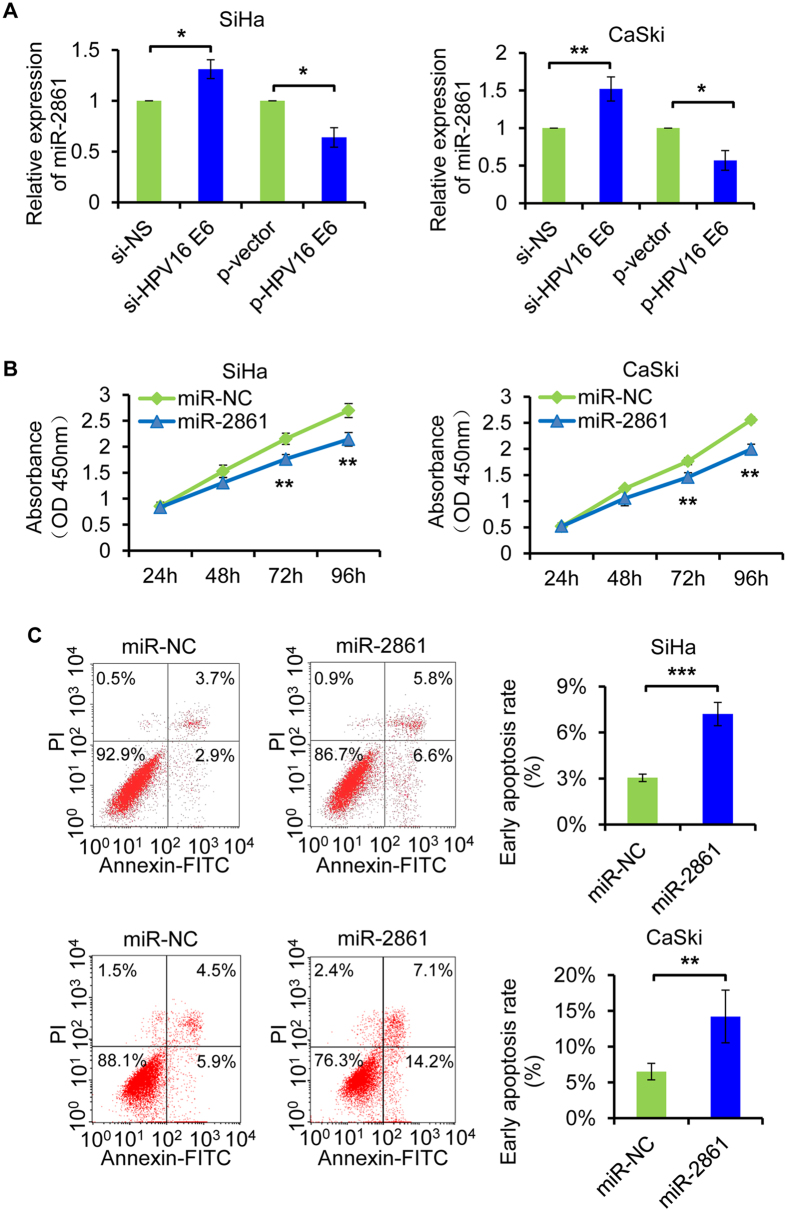
miR-2861 is regulated by HPV16 E6 and exerts tumor suppressive roles in cervical cancer SiHa and CaSki cells. (**A**) HPV16 E6 negatively regulates miR-2861 expression in both SiHa and CaSki cells. SiHa and CaSki cell lines were transfected with specific siRNA targeting HPV16 E6 or scrambled siRNA (left 2 lanes), and plasmid expression HPV16 E6 or empty vector (right 2 lanes) for 48 h, respectively. miR-2861 expression was assessed by qRT-PCR. (**B**) miR-2861 inhibits cell proliferation of SiHa and CaSki cells. Cells were plated in each well and transfected with miR-2861 or miR-NC. CCK8 assay was then performed at the time indicated. (**C**) miR-2861 enhances apoptosis of SiHa and CaSki cells. At 48 h after transfection with miR-2861 or miR-NC, SiHa and CaSki cells were collected for apoptosis assay using flow cytometry, respectively. **P < 0.01; ***P < 0.001.

Persistent infection with high-risk human papillomaviruses (HPVs) is known as the casual factor of cervical cancer^35^. Herein, we hypothesized whether HPV16 E6 also regulated miR-2861 in cervical cancer. As expected, knockdown of HPV16 E6 by HPV16 E6 siRNA transfection increased miR-2861 expression in two HPV16 positive cervical cancer cell lines, SiHa and CaSki. Contrarily, ectopic expression of HPV16 E6 by expression plasmid transfection reduced miR-2861 level in both SiHa and CaSki cells (Fig. 2A).

**Figure 3:**
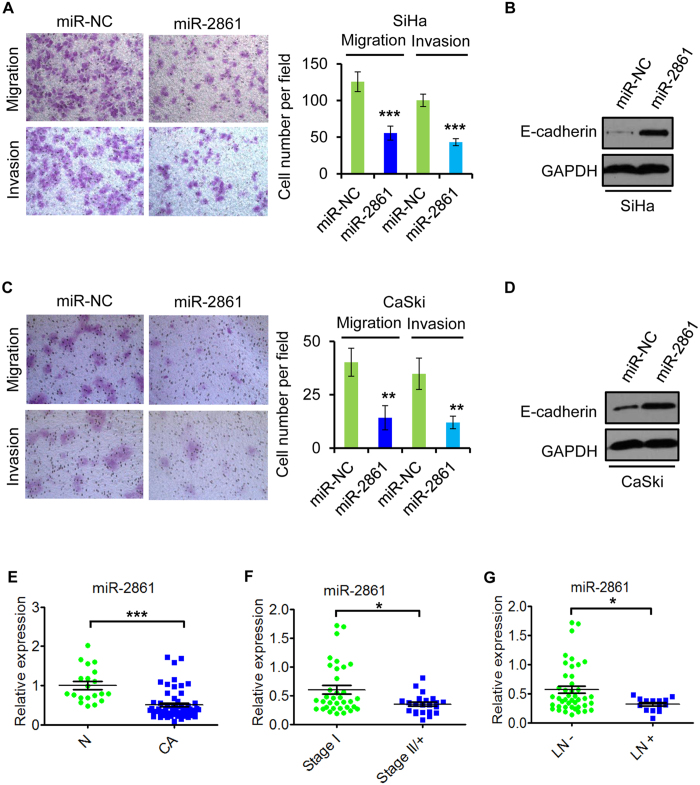
miR-2861 inhibits the migration and invasion of cervical cancer cells and is negatively associated with advanced tumor stage and lymph node metastasis. (**A**,**C**) miR-2861 inhibits the migration and invasion of SiHa (**A**) and CaSki (**C**) cells. At 24 h of posttransfection, cells were collected for migration (top) and matrigel invasion assays (bottom). Representative images are shown (magnification: ×200). The results are plotted as the average number of migrated or invasive cells from 5 randomly selected fields. Data are represented as the mean ± SD of three independent experiments. (**B**,**D**) miR-2861 increased E-cadherin expression level in SiHa (**B**) and CaSki (**D**) cells. 72 h after transfection, the protein level of E-cadherin was analyzed by Western blot. (**E**) miR-2861 is reduced in HPV16 positive cervical cancer tissues. qRT-PCR was performed to examine miR-2861 expression in 20 normal cervix and 57 HPV16 positive cervical cancer tissues. (**F**) miR-2861 is decreased in cervical cancer tissues with advanced tumor stage. qRT-PCR was used to assess miR-2861 expression. (**G**) miR-2861 is decreased in cervical cancer tissues with lymph node metastasis. qRT-PCR was used to analyze miR-2861 expression. *P < 0.05; **P < 0.01; ***P < 0.001.

To determine whether miR-2861 has potential biological roles in cervical cancer cells, we firstly performed gain-of-function studies using miR-2861 mimic to examine the effects on cell growth. The expression level of miR-2861 overexpression was validated by qRT-PCR (Supplementary Fig. S1C). miR-2861 overexpression suppressed the proliferation of both SiHa and CaSki cells compared to mimic negative control, as determined by a CCK8 assay (Fig. 2B). We next examined the impact of miR-2861 on apoptosis in SiHa and CaSki cells. The results revealed that enforced expression of miR-2861 significantly increased the rate of early apoptotis compared to control groups (Fig. 2C). To further demonstrate the actions of miR-2861, cell migration and matrigel invasion assay were performed. At 24 h post-transfection, the miR-2861 force-expressed SiHa (Fig. 3A) and CaSki (Fig. 3C) cells had a significant decrease in the migratory ability, respectively. Furthermore, overexpression of miR-2861 markedly reduced the invasive ability of SiHa (Fig. 3A) and CaSki (Fig. 3C) cells to invade through the matrigel-coated membrane towards the serum-containing medium in a chamber invasion assay, when compared with the negative control, respectively. Furthermore, the protein level of E-cadherin, a major critical molecule of EMT, was also increased with miR-2861 expression in both SiHa (Fig. 3B) and CaSki (Fig. 3D) cells. These results together suggest that miR-2861 acquire tumor suppressive potential through the dual advantages of weakened tumor cell proliferation and repressed cell invasion in cervical cancer SiHa and CaSki cells.

### miR-2861 is frequently reduced in HPV16 positive cervical cancer tissue samples

To further identify the potential role of miR-2861, we comparatively analyzed miR-2861 expression pattern of normal cervical tissues versus HPV16 positive cancer tissues by qRT-PCR. Compared with the 20 normal tissues, significant downregulation was observed in 84.2% (48/57) of cervical cancer samples (Fig. 3E). Meanwhile, we examined the correlation between miR-2861 levels and the clinicopathologic characteristics in the 57 cancer tissue samples with the associated clinical details (Table 1). We found that the expression of miR-2861 was reduced in those with the advanced tumor stage or lymph node metastasis (Fig. 3F,G), thus indicating that miR-2861 may act as a tumor suppressor in cervical cancer.

### miR-2861 directly targets EGFR, AKT2, and CCND1 in cervical cancer cells

**Figure 4:**
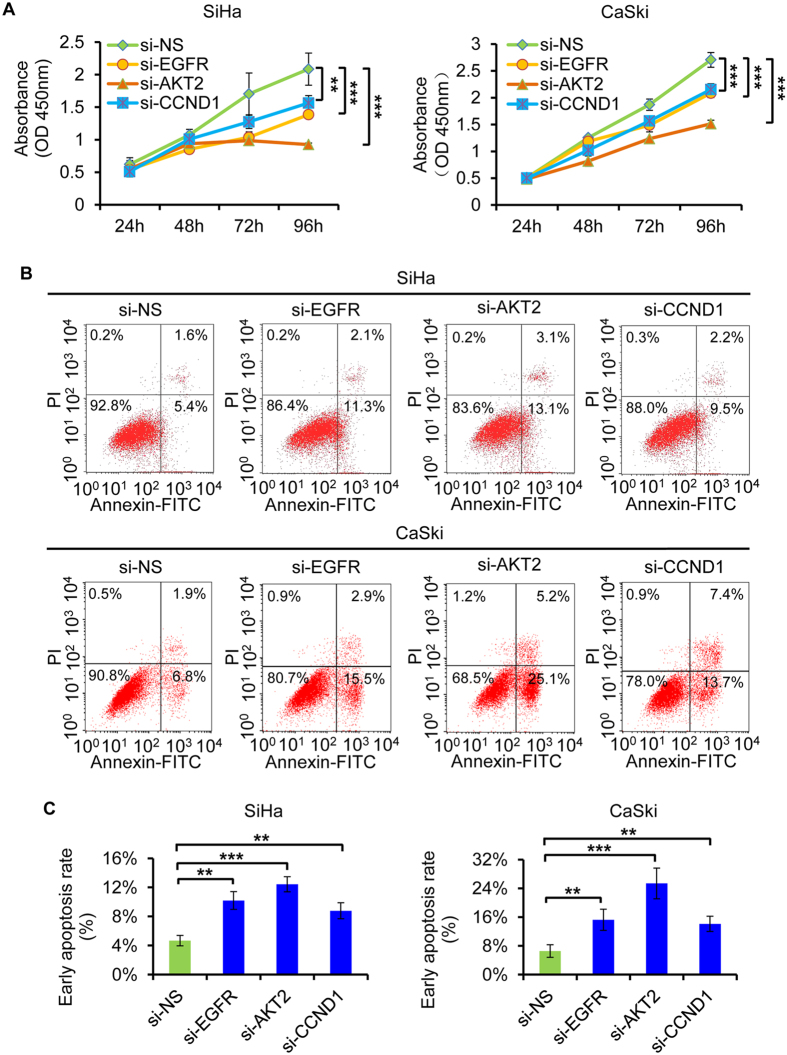
miR-2861 targets EGFR, AKT2, and CCND1 in cervical cancer cells. (**A**) Predicted EGFR, AKT2, and CCND1 3′ UTR binding sites for miR-2861. The alignment of the seed region of miR-2861 with EGFR, AKT2, and CCND1 3′UTR are shown. The mutated sites of targets are indicated in red. (**B**) EGFR 3′UTR is a target of miR-2861. pmiR-GLO luciferase construct containing a wt (left) or mutated (right) EGFR 3′UTR was cotransfected with miR-2861 or miR-NC in SiHa cells and the luciferase reporter assay was performed at 24 h posttransfection. (**C**) AKT2 3′UTR is a target of miR-2861. pmiR-GLO luciferase construct containing each wt (wt1, 2, 3, 4, and 5) or mutated (mut1, 2, 3, 4, and 5) AKT2 3′UTR was cotransfected with miR-2861 or miR-NC in SiHa cells, respectively. The luciferase assay was then performed. (**D**) CCND1 3′UTR is also a target of miR-2861. pmiR-GLO luciferase construct containing a wt (left) or mutated (right) CCND1 3′UTR was cotransfected with miR-2861 or miR-NC and the luciferase assay was performed. (**E**) miR-2861 overexpression decreased endogenous levels of EGFR, AKT2, and CCND1 proteins in SiHa and CaSki cells. SiHa and CaSki cells were transfected with miR-2861 or miR-NC for 72 h, respectively. EGFR, AKT2, and CCND1 expressions were assessed by Western blot. GAPDH was obtained as a loading control. Error bars, ± SD. *P < 0.05; **P < 0.01; NS, not significant.

To uncover the mechanisms by which miR-2861 suppressed cell growth and invasion, we search for the target genes of miR-2861. We adopted a TargerScan database searching method search and IPA analysis to identify putative targets. Among them, we found EGFR, AKT2, and CCND1 were the top candidates and of particular interesting because they have shown their essential roles in many cancers and are crucial components in the same signaling pathway^36^,^37^,^38^,^39^. The target prediction programs predicted that miR-2861 had one seed region that matched the 3′UTR of human EGFR (position of 3′UTR: 421-428); five seed regions matched the 3′UTR of human AKT2 (position of 3′UTR: site 1: 133–139; site 2: 435–442; site 3: 990-995; site 4: 1791–1798; site 5:1993-1998); and one seed region matched the 3′UTR of human CCND1 (position of 3′UTR: 2831–2837) (Fig. 4A).

To verify whether these genes were direct targets of miR-2861, a dual-luciferase reporter system was first employed. The EGFR 3′UTR, AKT2 3′UTR, and CCND1 3′UTR containing each miR-2861 binding site were cloned into the pmiRGLO control vector downstream of the firefly luciferase gene, respectively (Supplementary Fig. S2A–C). In case of EGFR, cotransfection of miR-2861 mimic with the EGFR 3′UTR-wt construct in SiHa cells resulted in a significant inhibition of luciferase activity compared with the negative control (Fig. 4B). Mutagenesis of the miR-2861 binding site with the EGFR 3′UTR abolished the ability of miR-2861 to regulate the luciferase expression (Fig. 4B, Supplementary Fig. S3A). For AKT2, cotransfection of miR-2861 with 5 separate AKT2 3′UTR-wt constructs was done, respectively. Upon miR-2861 overexpression, the luciferase activity of the site 1, 2, and 4 constructs were significantly inhibited compared with the control. However, there was no difference in the luciferase activities between the control and miR-2861 groups when the reporter construct carried the binding site 3 or 5 (Fig. 4C). Mutation of the miR-2861 binding sites within the AKT2 3′UTR constructs 1, 2 and 4 abolished the ability of miR-2861 to regulate the luciferase activity (Fig. 4C, Supplementary Fig. S3B). For CCND1, the luciferase assay showed that miR-2861 significantly led to the suppression of luciferase activity containing CCND1 3′UTR-wt (Fig. 4D). In contrast, miR-2861 had no effect on luciferase activity of a mutant 3′UTR of CCND1 construct (Fig. 4D, Supplementary Fig. S3C). In addition, miR-2861 overexpression reduced the endogenous protein levels of EGFR, AKT2, and CCND1 in both SiHa and CaSki cells compared with the controls, respectively. (Figure 4E, Supplementary Fig. S4A,B). Taken together, the above data suggest that miR-2861 is able to directly regulate EGFR, AKT2, and CCND1 expression in cervical cancer cells.

We also confirmed the experiment in HPV16 E6-expressing HaCaT (HaCaT-E6) cells. Either co-transfection of EGFR 3′UTR-wt, AKT2 3′UTR-wt1, AKT2 3′UTR-wt2, AKT2 3′UTR-wt4, or CCND1 3′UTR-wt reporter with miR-2861 mimic could cause repression of the luciferase activity compared to control group in the HaCaT-E6 cells (Supplementary Fig. S5). This was specific to miR-2861 binding since the activities were not affected when transfections were repeated with the mutations of these predicted miR-2861 binding sites in the 3′UTR (Supplementary Fig. S5). Similarly, there was no difference in the luciferase activities between the control and miR-2861 groups when co-transfected with AKT2 3′UTR-wt3 or AKT2 3′UTR-wt5 (Supplementary Fig. S5). These results indicate the direct effect of miR-2861 on EGFR/AKT2/CCND1 pathway in HPV16 E6-expressing cells.

### EGFR, AKT2, and CCND1 are involved in tumor suppression effects of miR-2861 in cervical cancer cells

**Figure 5:**
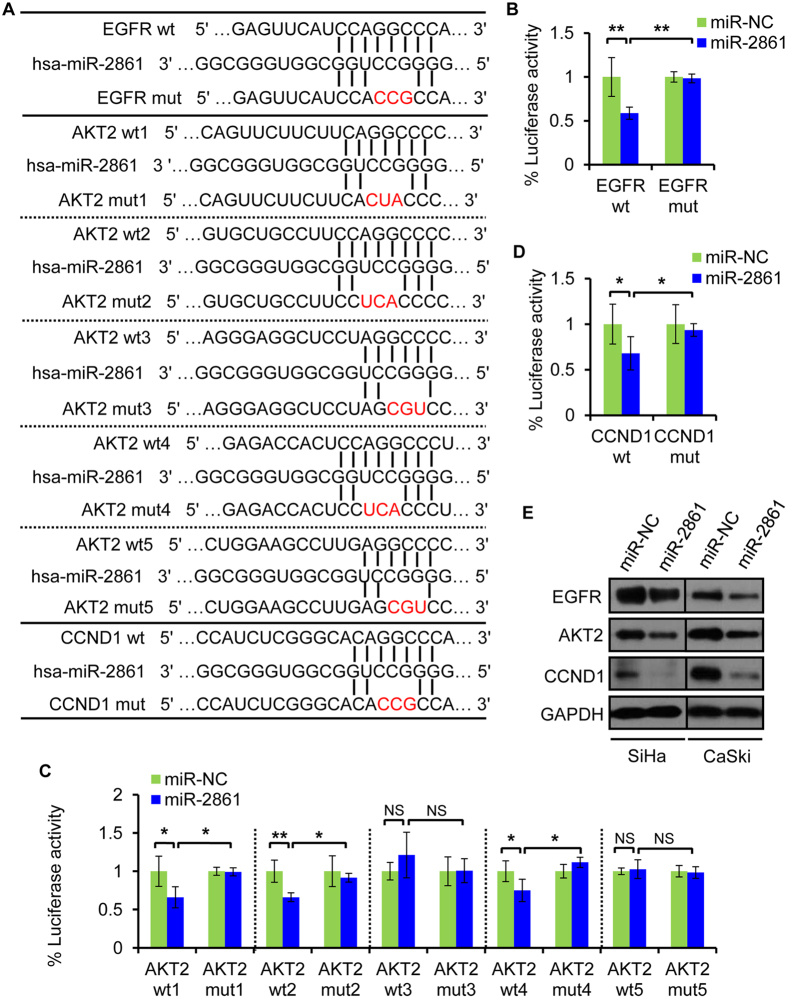
Knockdown of EGFR, AKT2, or CCND1 produces similar suppressive effects to that of miR-2861 overexpression in cervical cancer cells. (**A**) Knockdown of EGFR, AKT2, or CCND1 suppresses cell proliferation of both SiHa and CaSki cells. CCK8 assay was performed to determine the growth of SiHa and CaSki cells treated with si-EGFR, si-AKT2, si-CCND1, or si-NS. (**B**,**C**) Knockdown of EGFR, AKT2, or CCND1 enhances cell apoptosis of SiHa and CaSki cells. Apoptosis assay was determined in SiHa and CaSki cells at 48 h after transfection of si-EGFR, si-AKT2, si-CCND1, or si-NS. Representative images are shown (**C**) and early apoptosis rate are indicated (**B**). **P < 0.01; ***P < 0.001.

**Figure 6:**
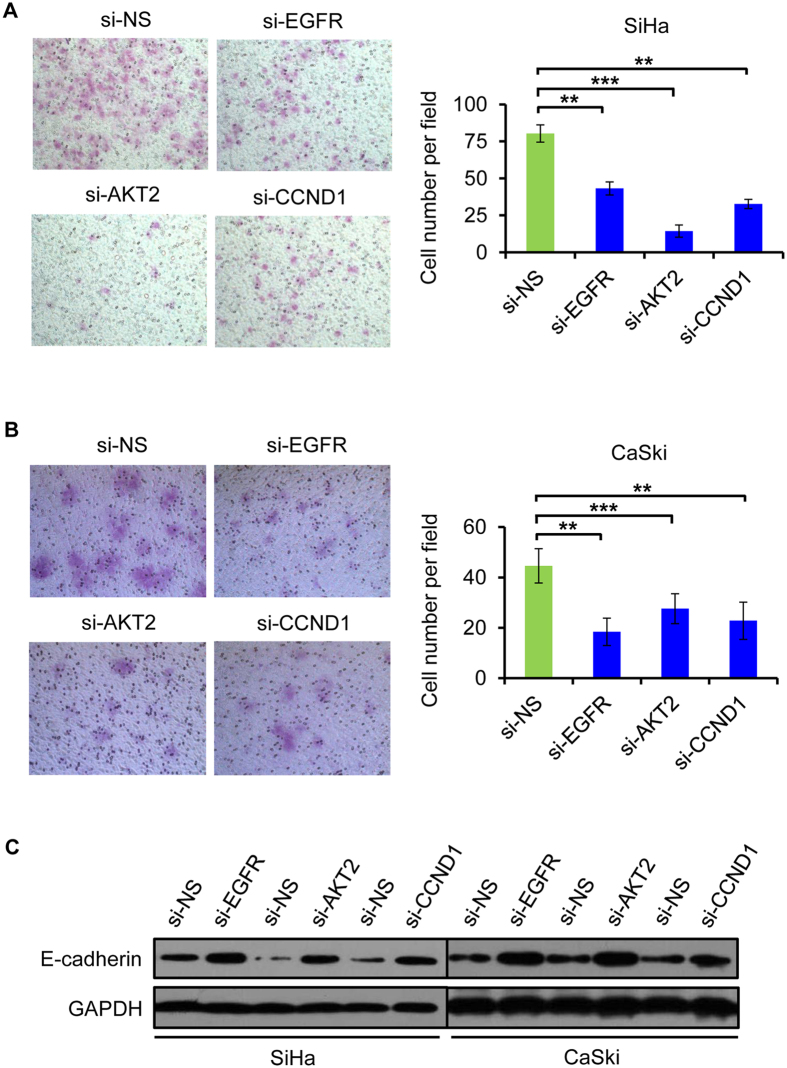
Knockdown of EGFR, AKT2, or CCND1 suppresses cell invasion and increased E-cadherin level in cervical cancer cells. (**A**,**B**) A matrigel invasion assay was performed in SiHa (**A**) and CaSki (**B**) cells transfected with si-EGFR, si-AKT2, si-CCND1, or si-NS. Representative images are shown (magnification: ×200). Error bars indicate ± SD. (**C**) Suppression of EGFR, AKT2, or CCND1 increased E-cadherin expression level in both SiHa and CaSki cells. 72 h after transfection, the protein level of E-cadherin was analyzed by Western blot. **P < 0.01; ***P < 0.001.

To explore the role of EGFR, AKT2, and CCND1 in miR-2861-regualted cell growth and invasion, we examined whether knockdown of these genes may mimic the effects of miR-2861 overexpression. SiHa and CaSki cells were transfected with specific siRNA duplex targeting either EGFR, AKT2, or CCND1, which resulted in a significant reduction in both mRNA and protein levels of the respective genes, respectively. Markedly, silencing of anyone of target genes led to suppress cell proliferation and induce apoptosis, phenolcopying the outcome of miR-2861 expression (Fig. 5A–C). Similar anti-invasion effects by EGFR, AKT2, or CCND1 silencing were also observed in SiHa and CaSki cells (Fig. 6A–C), which was in line with that of the miR-2861 overexpression.

**Figure 7:**
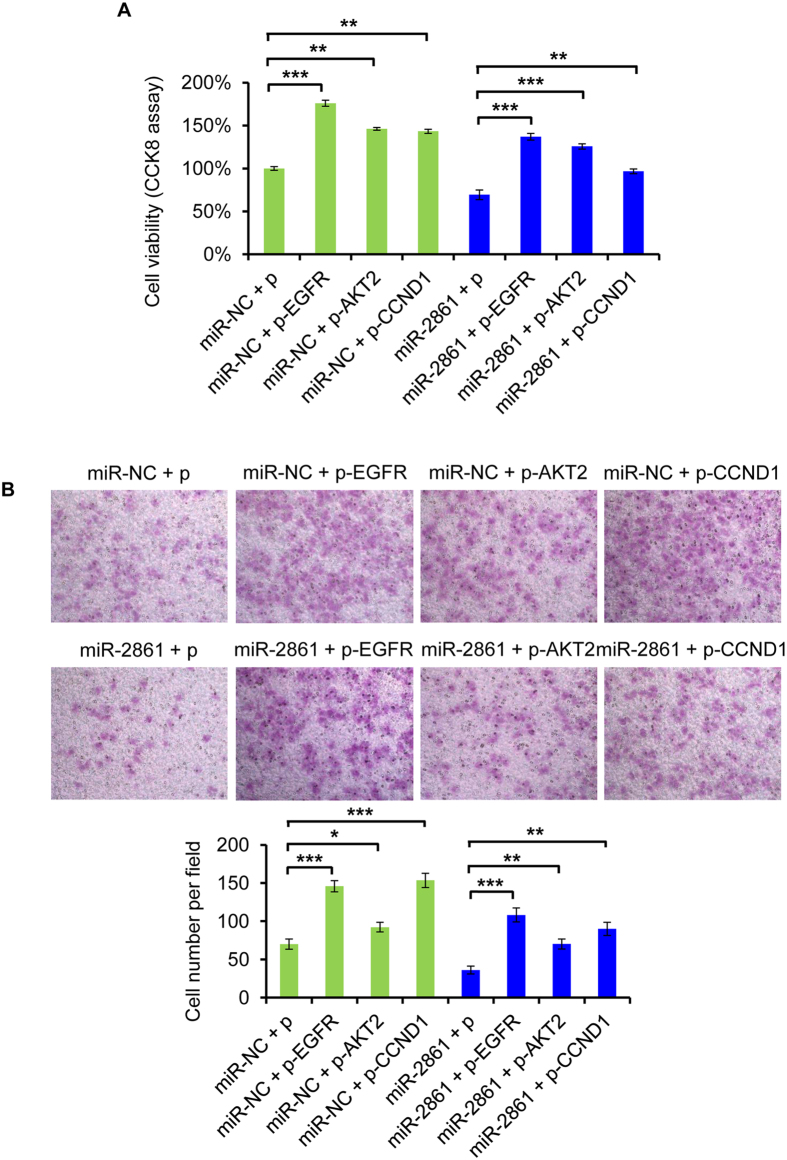
EGFR, AKT2, and CCND1 rescue miR-2861-induced cellular phenotypes in SiHa cells. Cells were cotransfected with EGFR, AKT2, CCND1 or empty vector and miR-2861 or miR-NC. (**A**) Overexpression of either EGFR, AKT2, or CCND1 recovers miR-2861-induced cell proliferation. CCK8 assay was assessed at 72 h after cotransfection. (**B**) Overexpression of either EGFR, AKT2, or CCND1 rescues miR-2861-induced inhibition of cell invasion. Representative images are shown (magnification: ×200). Error bars indicate ± SD. *P < 0.05; **P < 0.01; ***P < 0.001.

We subsequently examined whether EGFR, AKT2, and CCND1 could counteract the effect of miR-2861. We constructed EGFR, AKT2, and CCND1 overexpression vectors, respectively, which was cotransfected with miR-2861 mimic or miR-NC into SiHa cells. Notably, after cotransfected with the miR-2861, we found that either anyone of EGFR, AKT2, or CCND1 overexpression could rescued miR-2861-induced inhibition of cell growth and invasion in SiHa cells (Fig. 7A,B). Moreover, cotransfection with EGFR, AKT2, or CCND1 not only counterbalanced the tumor suppressor effects of miR-2861, but also promoted cancer cell growth and enhanced cell invasion ability (Fig. 7A,B). It may because of the complicated regulation network of these proteins. Though EGFR, AKT2, and CCND1 are regulated by multiple factors, out data suggest that miR-2861 exhibits tumor suppressor role by suppressing multiple targets including EGFR, AKT2, and CCND1 in HPV16 positive cervical cancer cells.

## Discussion

The abnormal constitutive expression of E6, as well as E7, is a key event in the malignant progression of HR-HPV infected cells and is associated with multiple alterations in viral and cellular pathways^40^. Although the best-known property of the HR-HPV E6 is its ability to bind and degrade the tumor suppessor TP53 via a proteasome pathway, but much detail remains to be learned. In the past 10 years, dysregulation of miRNAs has been shown to be a common event that can control nearly all biological processes such as tumorigenesis, cell growth, cell invasion, angiogenesis, and chemoresistance in cancer development and progression^41^,^42^.

In this study, we determined the dysregulated expression profiles of miRNAs with HPV16 E6 overexpression using miRNA microarray. We identified 59 miRNAs that were differentially expressed in HPV16 E6 overexpressed HEK293T cells compared to the corresponding vector control cells. We also validated 15 miRNAs in two cell lines, p53-wild HEK293T cells and p53-mutant HaCaT cells. 3 of the 15 miRNAs showed significant difference in both two cell lines, and miR-2861 was the only down-regulated one in response to HPV16 E6 overexpression. The miR-2861 gene is located on chromosome 2 and conserved in the human sequence. miR-2861 promoted BMP2-induced osteoblast differentiation by targeting histone deacetylase 5 (HDAC5)^43^,^44^. Upregulation of miR-2861 was found in palillary thyroid carcinoma with lymph node metastasis^45^, while downregulation of miR-2861 was shown in basal cell carcinoma^46^. To date, the role of miR-2861 and dysregulated miR-2861 in cancer are still uncertain. Here, we validated the expression pattern and biological actions of miR-2861 in two HPV16 positive cervical cancer SiHa and CaSki cells, and showed that HPV16 E6 suppressed miR-2861 expression and gain-of-function of miR-2861 inhibited cell proliferation and invasion, and promoted apoptosis in cervical cancer cells. Furthermore, we analyzed the clinical significance of abnormal miR-2861 expression in cervical cancer and found that miR-2861 was significantly downregulated in cervical cancer tissues that was negatively associated with advanced tumor stage and lymph node metastasis. Taken together, our data provided a comprehensive understanding of the tumor suppressor role of miR-2861 in HPV16-induced cervical cancer.

To elucidate the regulatory mechanisms of miR-2861 in cervical cancer, we identified, for the first time, EGFR, AKT2, and CCND1 as the targets of miR-2861 in both SiHa and CaSki cells. EGFR is overexpressed in a wide variety of solid tumors, including cervical cancer^47^, contributes to growth activity and tumor survival, and is therefore a therapeutic target in tumors^48^. AKT2, a homolog of the v-akt oncogene, is the member of the AKT family of proteins (AKT1, AKT2, AKT3) which are activated by the PI3K pathway^49^,^50^. Also, PI3K/AKT signaling pathway is the major link between oncogenic EGFR and downstream pro-survival molecules and one of the most frequently activated pathways in human cancers^51^,^52^,^53^. Moreover, activation of AKT has been demonstrated to promote cancer cell proliferation and survival signals via upregulation of CCND1^36^,^54^. Here, we demonstrated that miR-2861 suppressed all the three members of the EGFR/AKT2/CCND1 signaling pathway by directly binding to the 3′UTR of EGFR, AKT2, and CCND1, and found that silencing anyone of EGFR or AKT2 or CCND1 could inhibit the tumor properties of cervical cancer cells, similar to that of miR-2861 overexpression. Meanwhile, overexpression of EGFR, AKT2, and CCND1 eliminate the effect of miR-2861 on cervical cancer cells. Therefore, the activation of the EGFR/AKT2/CCND1 signaling pathway induced by HPV16 E6-deprivated miR-2861 expression in cervical cancer cells may, at least partially, explain the tumor suppression effects of miR-2861 in cervical cancer.

Here, we showed that HPV16 E6 is able to downregulate miR-2861 expression level. We also demonstrated that overexpression of miR-2861 resulted in the suppression of EGFR/AKT2/CCND1 pathway in cervical cancer cells. In summary, we delineates a novel regulatory network employing HPV16 E6, miR-2861, and EGFR/AKT2/CCND1 signaling pathway to fine-tune proliferation, apoptosis, and invasion in cervical cancer cells. This understanding of unique molecular pathway, HPV16 E6/miR-2861/EGFR/AKT2/CCND1, may provide the novel insight into the exploration of additional strategies for cervical cancer therapy in the future.

## Materials and Methods

### MicroRNA microarray

Total RNA was extracted from two groups of HEK293T cells on day 2 following HPV16 E6 overexpression or not, with three independent biological samples per group, using total RNA purification kit (LC Sciences, Houston, TX, USA) according to the manufacturer’s instructions. The quality and quantity of the RNA samples were assessed by standard electrophoresis and spectrophotometer methods. miRNA microarray assay was carried out using the μParaflo microfluidic technology according to the manufacturer’s protocol (LC Sciences). Briefly, 5 μg of total RNA was labeled and hybridized to each miRNA microarray (μParaflo miRNA microarray version 19.0, LC Sciences). After RNA hybridization, tag-conjugating Cy3 dye was circulated through the microfluidic chip for dye staining. Hybridization images were collected with a GenePix 4000B laser scanner (Molecular Device, CA, USA) and digitized with Array-Pro image analysis software (Media Cybernetics, MD, USA). The data were analyzed by first subtracting the background and then normalizing the signals using a LOWESS filter (Locally-weighted Regression)^55^. The ratio of the two groups of detected signals (log_2_ (p-HPV16 E6/p-vector)) and *P*-values of the t-test were calculated. Differentially expressed miRNAs were defined when the *P*-value was less than 0.05 and fold changes ≥2.

### Cell culture and transfection

All cells (human embryonic kidney 293-T cell line HEK293T, human normal epithelial cell line HaCaT, HPV16 positive cervical cancer cell lines SiHa and CaSki were seeded and grown in DMEM medium supplemented with 10% fetal bovine serum (Gibco, NY, USA), and maintained at 37 °C in an atmosphere of 5% CO_2._ The HaCaT cells were transfected with HPV16 E6 expression vector and selected for 10 days with G-418 at 300 μg/ml to derive HPV16 E6-expressing HaCaT-E6 cells. All plasmid transfections were performed by using X-tremeGENE HP DNA transfection reagent (Roche), as suggested by the manufacturer. All miRNA or siRNA transfections were performed by using DharmaFECT1 transfection reagent (Dharmacon) as per manufacturer’s protocol.

HPV16 E6 CDS, EGFR CDS, AKT2 CDS, and CCND1 CDS were chemically synthesized and subcloned to pcDNA 3.1+/vector using Bam H1/XhoI sites by GenScript (Nanjing, China), respectively. miR-2861 mimic and mimic negative control (miR-NC) were purchased from Dharmacon (CO, USA). Specific siRNA sequences targeting HPV16 E6, EGFR, AKT2, and CCND1 were designed and synthesized by GenePharma (Shanghai, China), respectively.

### Clinical tissue specimens and evaluation of HPV status

Fresh cervical cancer tissue samples were obtained from 57 patients with cervical cancer who underwent surgical resections at Department of Gynecologic Oncology, Women’s Hospital, School of Medicine, Zhejiang University, China. The clinical data were obtained from medical records with the hospital and clinicopathological characteristics of these patients are collected. Samples of normal cervical tissues were obtained from 20 patients who underwent hysterectomy because of benigh gynecological diseases. These samples were immediately frozen in liquid nitrogen and used for validation. The study was approved by the ethical board of the Women’s Hospital of Zhejiang University and written informed consent was obtained from all of the patients. All the experiments were carried out in accordance with the approved guidelines.

High-risk HPV DNA was firstly confirmed by Hybrid Capture 2 assay (HC2, Digene, Gaithersburg, MD) for all these patients before operation. The exfoliated cells were obtained after the primary HPV testing, and genomic DNA was extracted. Flow-through hybridization (HybriMax) was used for HPV genotyping. Normal tissue samples without HPV infection were precisely determined by PCR using L1 consensus primers MY09 and MY11^56^.

### RNA extraction, reverse transcription, and quantitative real-time PCR

Total RNA (except for microarray) was extracted from prepared cell lines or cervical tissues using TRizol Reagent (Invitrogen, CA, USA) and cDNA was synthesized with the PrimiScript RT reagent kit (TaKaRa, Shiga, Japan). Quantitative real-time PCR (qPCR) was performed using the SYBR Premix Ex Taq (TaKaRa), and PCR-specific amplification was conducted in the Applied Biosystems 7900HT real-time PCR mechine (ABI, CA, USA). For miRNA quantification, stem-loop RT-PCR was performed. The primers of miRNAs were all purchased from Ribobio, Guangzhou, China. U6 snRNA and GAPDH were used as endogenous control for miRNA and mRNA, respectively. The relative expression of miRNAs or genes was calculated with the 2^−(∆∆Ct)^ method^57^.

### Dual luciferase reporter gene assay

PmirGLO-Dual-Luciferase reporter vector (7350 bp, Promega, WI, USA) was used to confirm the function of putative miR-2861 binding sites in the EGFR 3′UTR, AKT2 3′UTR, and CCND1 3′UTR. Primers were designed for each potential miR-2861 binding sequence of EGFR 3′UTR, AKT2 3′UTR, and CCND1 3′UTR, respectively, and then clone into the Sac I/Xba I sites of pmirGLO-Dual-Luciferase reporter vector. The reconstructed plasmids were confirmed by sequencing, and named pmirGLO/EGFR-wt, pmirGLO/AKT2-wt1, pmirGLO/AKT2-wt2, pmirGLO/AKT2-wt3, pmirGLO/AKT2-wt4, pmirGLO/AKT2-wt5, and pmirGLO/CCND1-wt. We also commercially synthesized mutant reporter constructs by mutating three nucleotides of each potential miR-2861 binding site, and termed pmirGLO/EGFR-mut, pmirGLO/AKT2-mut1, pmirGLO/AKT2-mut2, pmirGLO/AKT2-mut3, pmirGLO/AKT2-mut4, pmirGLO/AKT2-mut5, and pmirGLO/CCND1-mut.

Cells of 90% confluence were seeded in triplicate in 96-well plates. The wild-type (WT) or mutant reporter constructs (Mut) were cotransfected into SiHa cells in the 96-well plates with 50 nmol/L miR-2861 or 50 nmol/L miR-NC by using lipofectamine 2000 (Invitrogen, CA, USA), respectively. Repoter gene assays were performed 24 h posttransfection using the Dual Luciferase Reporter Assay Kit (Promega) according to the manufacturer’s instructions. Firefly luciferase activity values were normalized for transfection efficiency using the corresponding Renilla luciferase activity. Three independent experiments were performed.

### Western blot analysis

72 h after transfection, cell protein lysates were separated in 8% or 10% SDS-PAGE gel, followed by subsequently transferred to polyvinylidene difluoride membrane (PVDF). Western blot analysis was performed with monoclonal anti-p53 (Santa cruz), anti-EGFR (Abcam), anti-AKT2 (Abcam), anti-CCND1 (Abcam), and anti-E-cadherin (Abcam) antibodies. Anti-GAPDH antibody (Santa cruz) was used as an internal control. The membrane was washed and incubated with horseradish peroxidase (HRP)-conjugated secondary antibodies (Cell Signaling Technology, USA). Complexes were visualized with SuperSignal West Pico Chemiluminescent Substrate (Pierce) and the expression levels of these proteins were evaluated by Quantity One software.

### Cell proliferation assay

SiHa and CaSki cells were plated on 96-well plates at 4000~5000 cells/well, transfected with miRNA mimic, siRNA, or expression plasmid, respectively. CCK8 assay was performed at 24 h, 48 h, 72 h, and 96 h post transfection. The absorbance of the samples was measured with a spectrophotometer reader at 450 nm. Each assay was performed in triplicate and repeated three times independently.

### Cell apoptosis assay

An annexin V-fluorescein insothiocyanate (FITC) & PI apoptosis detection kit (Biouniquer, Nanjing, China) and flow cytometry (BD Biosciences, CA, USA) were used to detect the percentage of early apoptosis. The measurement was repeated three times independently.

### Cell migration and invasion assays

Cell migration assays were performed using modified Boyden Chambers (Transwells, Corning Incorporated, NY, USA) containing uncoated Transwell polycarbonate membrane fliters with 8-μm pores in 24-well plates. SiHa and CaSki cells were transfected with 50 nm miRNA or 50 nm siRNA, respectively. After 24 h, cell viability was confirmed by Trypan blue assay prior to apply for the migration assay. Then, cells with similar viability in each group were added to the upper chamber of each migration well, and 500 μl of DMEM with 10% FBS was added to the lower part of the chamber and allowed to migrate for 16 h. After gentle removal of the non-migratory cells from the filter surface of the upper chamber, cells that migrated to the lower side were fixed and stained with 1% crystal violet solution for 1 h. Then, the number of the cells migrating to the lower surface was determined microscopically by counting 5 random visual fields per well. Cell invasion assays were carried out using the chambers containing transwell precoated Matrigel membrane filter inserts with 8-μm pores in the 24-well plates at 1 × 10^5^ cells per well (BD Biosciences, USA). All assays were performed three independent times.

### Statistical analysis

Statistical analyses were performed by SPSS 21.0 statistical software (SPSS, Inc., USA) and GraphPad Prism 5 (GraphPad software, Inc., CA, USA). Comparisons between two groups were performed by Student’s t test. Statistical significance was defined as *P* < 0.05. *P < 0.05; **P < 0.01; ***P < 0.001; NS, not significant.

## Additional Information

**How to cite this article**: Xu, J. *et al*. miR-2861 acts as a tumor suppressor via targeting EGFR/AKT2/CCND1 pathway in cervical cancer induced by human papillomavirus virus 16 E6. *Sci. Rep.* **6**, 28968; doi: 10.1038/srep28968 (2016).

## Supplementary Material

Supplementary Information

## Figures and Tables

**Table 1:** Clinicopathological characteristics and miR-2861 expression in cervical cancer patients.

Clinicopathological factors	Number of cases (n = 57) (%)	Mean expression of miR-2861 (range) (2^−△△CT^ ± SD)	*P* value
Age (years)			0.543^a^
<35	7 (12.3%)	0.43 ± 0.30 (0.20–1.09)	
≥35	50 (87.7%)	0.51 ± 0.39 (0.08–1.71)	
Tumor stge			0.026^a^
I B	35 (61.4%)	0.60 ± 0.43 (0.19–1.71)	
IIA	22 (38.8%)	0.35 ± 0.17 (0.08–0.81)	
Differentiation			0.347^a^
Well/Moderate	49 (86.0%)	0.53 ± 0.39 (0.14–1.71)	
Poor	8 (14.0%)	0.34 ± 0.15 (0.08–0.55)	
Tumor size (cm)			0.213^a^
<4	51 (89.5%)	0.53 ± 0.39 (0.14–1.71)	
≥4	6 (10.5%)	0.30 ± 0.14 (0.08–0.46)	
SCC (ng/ml)			0.884^a^
<4	49 (86.0%)	0.51 ± 0.40 (0.08–1.71)	
≥4	8 (14.0%)	0.44 ± 0.20 (0.20–0.81)	
Deep stromal invasion			0.759^a^
<2/3	33 (57.9%)	0.51 ± 0.37 (0.08–1.71)	
≥2/3	24 (42.1%)	0.50 ± 0.38 (0.19–1.70)	
Vsucular involvement			0.222^a^
Negative	37 (64.9%)	0.54 ± 0.39 (0.14–1.71)	
Positive	20 (35.1%)	0.43 ± 0.34 (0.08–1.58)	
Lymph node metastasis			0.047^a^
Negative	43 (75.4%)	0.56 ± 0.41 (0.14–1.71)	
Positive	14 (24.6%)	0.32 ± 0.11 (0.08–0.47)	

^a^*P* value when expression levels were compared using the Mann - Whitney test.
