# Supplementary material for: miR-2861 acts as a tumor suppressor via targeting EGFR/AKT2/CCND1 pathway in cervical cancer induced by human papillomavirus virus 16 E6
Source: Sci Rep. 2016 Jul 1;6:28968. doi: 10.1038/srep28968 (PMC4929448; doi:10.1038/srep28968)
Supplement: Supplementary Information [file srep28968-s1.pdf]

# miR-2861 acts as a tumor suppressor via targeting EGFR/AKT2/CCND1 pathway in cervical cancer induced by human papillomavirus virus 16 E6

Junfen Xu[1], Xiaoyun Wan[1], Xiaojing Chen[2], Yifeng Fang[3], Xiaodong Cheng[1], Xing Xie[1], Weiguo Lu[1,*]

[1] Department of Gynecologic Oncology, Women's Hospital, School of Medicine, Zhejiang University, Hangzhou, Zhejiang, 310006, China;

[2] Women's Reproductive Health Laboratory of Zhejiang Province, Women's Hospital, School of Medicine, Zhejiang University, Hangzhou, Zhejiang, 310006, China;

[3] Department of General Surgery, Sir Run Run Shaw Hospital, School of Medicine, Zhejiang University, Hangzhou 310016, China;

* Corresponding author

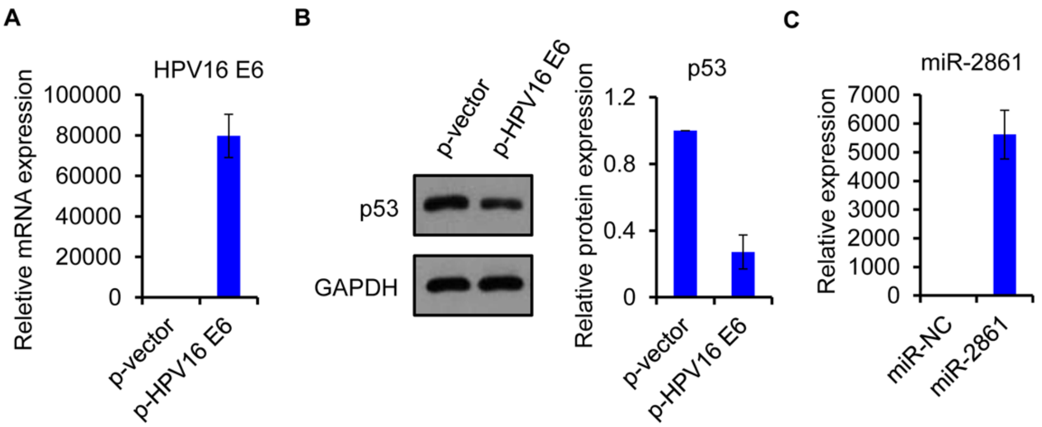


**Figure S1. Transfection levels of expression plasmid of HPV16 E6 and miR-2861.** (A) At 48h after transfection with HPV16 E6 expression plasmid (2μg) in HEK293T cells, the expression of HPV16 E6 level was determined by qRT-PCR. (B) At 72h after transfection with HPV16 E6 expression plasmid in HEK293T cells, p53 expression level was evaluated by Western blot. (C) At 48h after transfection with miR-2861 mimic (50 nM) in SiHa cells, the expression of miR-2861 was determined by qRT-PCR.

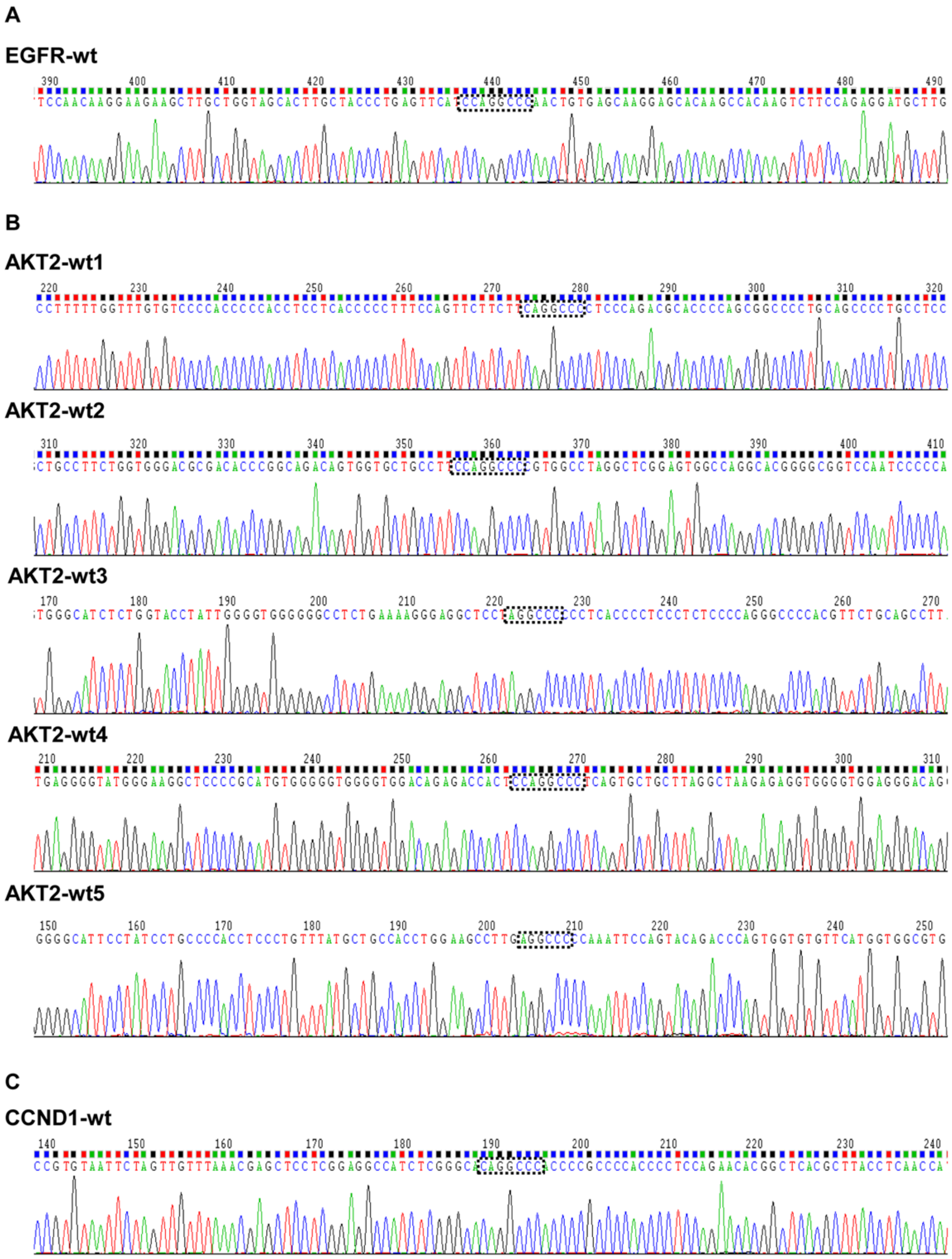


**Figure S2. Sequences of EGFR, AKT2, and CCND1 3'UTR containing miR-2861 binding site.** (A) Sequence of EGFR 3'UTR containing miR-2861 binding site. (B) Sequences of 5 AKT2 3'UTR wild type plasmids, each of which contains one binding site of miR-2861. (C) Sequence of CCND1 3'UTR containing miR-2861 binding site. Blank box indicates the binding site of miR-2861.

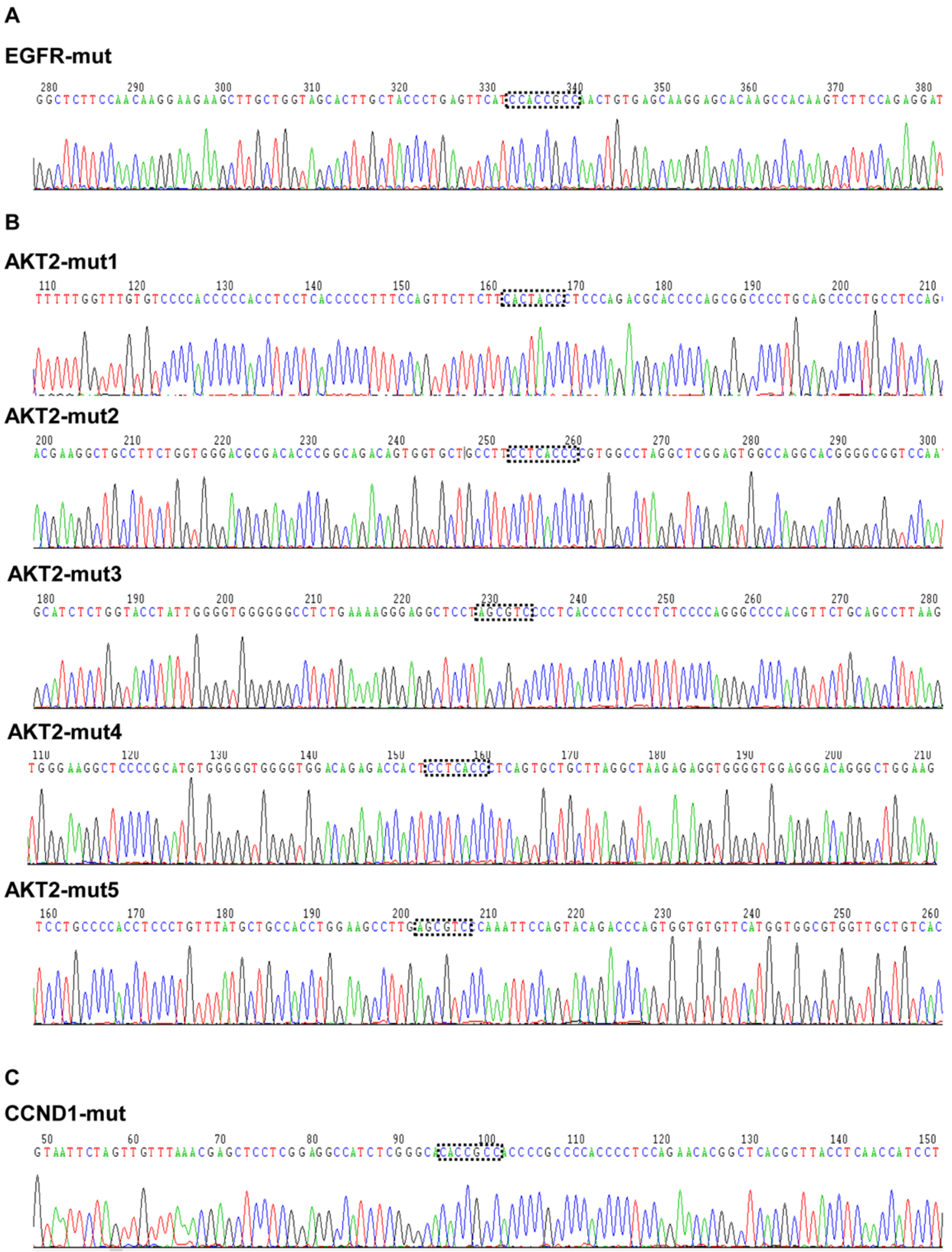


**Figure S3. Sequences of mutant EGFR, AKT2, and CCND1 3'UTR.** (A-C) The sequences of miR-2861 binding sites on EGFR, AKT2, and CCND1 3'UTR are mutant. Blank box indicates the mutant binding site of miR-2861.

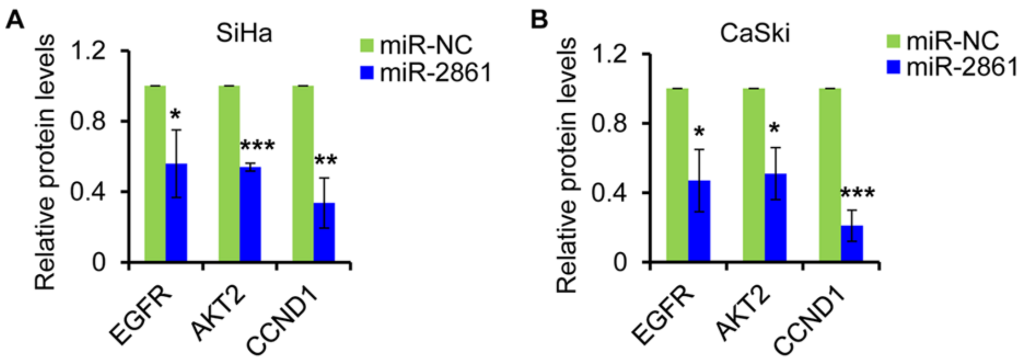


**Figure S4. miR-2861 targets EGFR, AKT2, and CCND1 in cervical cancer cells.** (A and B) Graph depicts the relative expression of EGFR, AKT2, and CCND1 protein levels in response to miR-2861 overexpression in cervical cancer SiHa (A) and CaSki (B) cells. Data are normalized to GAPDH levels and expressed as fold change from the control (miR-NC). Error bars indicate ± SD. * $P<0.05$; ** $P<0.01$; *** $P<0.001$.

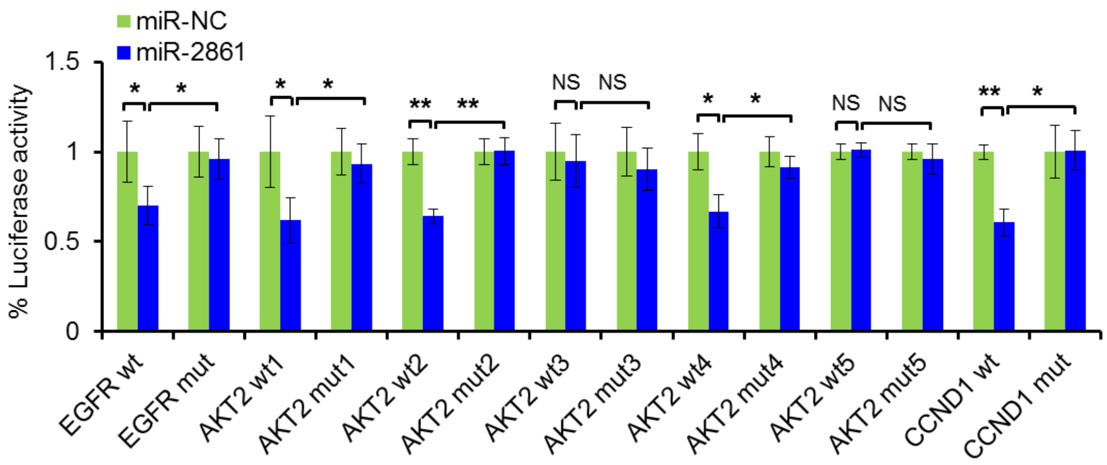


**Figure S5. miR-2861 targets EGFR, AKT2, and CCND1 in HPV16 E6-expressing HaCaT (HaCaT-E6) cells.** pmiR-GLO luciferase construct containing each wt (EGFR wt, AKT2 wt1, AKT2 wt2, AKT wt3, AKT wt4, AKT2 wt5, or CCND1 wt) or mutated (EGFR mut, AKT2 mut1, AKT2 mut2, AKT2 mut3, AKT2 mut4, AKT2 mut5, or CCND1 mut) 3'UTR was cotransfected with miR-2861 or miR-NC in HaCaT-E6 cells, respectively. The luciferase assay was then performed. Error bars, ± SD. * $P<0.05$; ** $P<0.01$; NS, not significant.

**Table S1.** Differentially expressed miRNAs between HPV16 E6 and vector control groups in HEK293T cells

| Accession No. | Reporter Name | p-value | $\log_2$ (fold change) |
|---|---|---|---|
| MIMAT0004485 | hsa-let-7e-3p | 4.22E-03 | 4.94 |
| MIMAT0022296 | hsa-miR-5588-3p | 6.63E-03 | 3.17 |
| MIMAT0010367 | hsa-miR-764 | 7.03E-03 | 5.70 |
| MIMAT0003273 | hsa-miR-605 | 7.16E-03 | 5.31 |
| MIMAT0019808 | hsa-miR-4707-3p | 8.68E-03 | 3.63 |
| MIMAT0003879 | hsa-miR-758-3p | 1.02E-02 | 3.11 |
| MIMAT0019979 | hsa-miR-4800-3p | 1.04E-02 | 2.53 |
| MIMAT0019722 | hsa-miR-4655-3p | 1.06E-02 | 3.16 |
| MIMAT0004482 | hsa-let-7b-3p | 1.19E-02 | 4.03 |
| MIMAT0019820 | hsa-miR-4713-5p | 1.45E-02 | 3.12 |
| MIMAT0013802 | hsa-miR-2861 | 1.47E-02 | -0.81 |
| MIMAT0010195 | hsa-let-7a-2-3p | 1.52E-02 | 3.83 |
| MIMAT0022939 | hsa-miR-939-3p | 1.54E-02 | 3.44 |
| MIMAT0002815 | hsa-miR-432-3p | 1.64E-02 | 3.25 |
| MIMAT0002170 | hsa-miR-412 | 1.80E-02 | 2.62 |
| MIMAT0018065 | hsa-miR-3646 | 1.82E-02 | 4.37 |
| MIMAT0022726 | hsa-miR-1306-5p | 1.84E-02 | 1.80 |
| MIMAT0009203 | hsa-miR-449b-3p | 1.88E-02 | 4.05 |
| MIMAT0005921 | hsa-miR-1267 | 1.89E-02 | 3.43 |
| MIMAT0002173 | hsa-miR-483-3p | 1.98E-02 | 2.25 |
| MIMAT0019940 | hsa-miR-4436b-5p | 1.98E-02 | 5.08 |
| MIMAT0004986 | hsa-miR-943 | 2.11E-02 | 3.52 |
| MIMAT0016906 | hsa-miR-4274 | 2.16E-02 | 2.17 |
| MIMAT0004813 | hsa-miR-411-3p | 2.18E-02 | 4.20 |
| MIMAT0022732 | hsa-miR-3191-5p | 2.18E-02 | 2.79 |
| MIMAT0018085 | hsa-miR-3663-3p | 2.24E-02 | 2.67 |
| MIMAT0016909 | hsa-miR-4279 | 2.30E-02 | 2.21 |
| MIMAT0019212 | hsa-miR-3160-5p | 2.33E-02 | 3.74 |

| | | | |
|---|---|---|---|
| MIMAT0007884 | hsa-miR-1910 | 2.36E-02 | 3.43 |
| MIMAT0019957 | hsa-miR-4787-3p | 2.36E-02 | 2.91 |
| MIMAT0025845 | hsa-miR-6716-3p | 2.47E-02 | 3.73 |
| MIMAT0019789 | hsa-miR-4695-3p | 2.53E-02 | 4.14 |
| MIMAT0022720 | hsa-miR-1304-3p | 2.59E-02 | 3.50 |
| MIMAT0004703 | hsa-miR-335-3p | 2.63E-02 | 4.78 |
| MIMAT0003300 | hsa-miR-631 | 2.69E-02 | 2.96 |
| MIMAT0004677 | hsa-miR-34c-3p | 2.90E-02 | 5.24 |
| MIMAT0004607 | hsa-miR-138-1-3p | 3.03E-02 | 3.01 |
| MIMAT0019219 | hsa-miR-3619-3p | 3.03E-02 | 3.01 |
| MIMAT0019877 | hsa-miR-3591-3p | 3.32E-02 | 3.01 |
| MIMAT0019226 | hsa-miR-3150b-5p | 3.34E-02 | 3.18 |
| MIMAT0003888 | hsa-miR-766-3p | 3.35E-02 | 1.75 |
| MIMAT0003298 | hsa-miR-629-3p | 3.42E-02 | 2.78 |
| MIMAT0004615 | hsa-miR-195-3p | 3.49E-02 | 3.97 |
| MIMAT0004946 | hsa-miR-744-3p | 3.55E-02 | 2.87 |
| MIMAT0001639 | hsa-miR-409-3p | 3.60E-02 | 4.35 |
| MIMAT0009451 | hsa-miR-1976 | 3.62E-02 | 2.33 |
| MIMAT0018350 | hsa-miR-3935 | 3.93E-02 | 2.62 |
| MIMAT0004804 | hsa-miR-615-5p | 4.00E-02 | 3.02 |
| MIMAT0019854 | hsa-miR-4731-3p | 4.01E-02 | 2.97 |
| MIMAT0000437 | hsa-miR-145-5p | 4.06E-02 | 2.51 |
| MIMAT0000269 | hsa-miR-212-3p | 4.08E-02 | 3.53 |
| MIMAT0004819 | hsa-miR-671-3p | 4.09E-02 | 3.22 |
| MIMAT0005573 | hsa-miR-1225-3p | 4.10E-02 | 2.69 |
| MIMAT0000450 | hsa-miR-149-5p | 4.26E-02 | 2.40 |
| MIMAT0019857 | hsa-miR-4733-5p | 4.26E-02 | 3.31 |
| MIMAT0021024 | hsa-miR-5002-3p | 4.34E-02 | 3.39 |
| MIMAT0000227 | hsa-miR-197-3p | 4.62E-02 | 1.60 |
| MIMAT0003310 | hsa-miR-640 | 4.80E-02 | 2.85 |
| MIMAT0019904 | hsa-miR-4758-3p | 4.88E-02 | 4.25 |
